# Effects of Sedation, TEmperature and Pressure After Cardiac Arrest and REsuscitation on Major Adverse Kidney Events (STEPCARE‐MAKE): A Protocol for a Pre‐Planned Sub‐Study of a Randomized Clinical Trial

**DOI:** 10.1111/aas.70276

**Published:** 2026-06-15

**Authors:** Keski‐Keturi M, Niemelä V.H, Young P, Nielsen N, Wise M.P, Reinikainen M, Bass F, Lilja G, Dankiewicz J, Hammond N, Hästbacka J, Levin H, Moseby‐Knappe M, Saxena M, Tiainen M, Ceric A, Kamp C.B, Sillassen C.D.B, Alexandersson D, Schmidbauer S, Tirkkonen J, Oksanen T, Säkkinen H, Bendel S, Düring J, Lybeck A, Johnsson J, Unden J, Lundin A, Kåhlin J, Grip J, Lotman E, Romundstad L, Seidel P, Stammet P, Graf T, Mengel A, Leithner C, Nee J, Druwé P, Ameloot K, McGuigan P.J, White J, Govier M, Ostermann M, Hopkins P, Proudfoot A, Handslip R, Pogson D, Jackson P, Nichol A, Haenggi M, Hilty M.P, Iten M, Schrag C, Nafi M, Joannidis M, Robba C, Belohlavek J, Rob D, Arabi Y, Al‐Fares A, Yew Woon C, Aneman A, Stewart A, Arnott C, Ramanan M, Panwar R, Delaney A, Reade M, Venkatesh B, Taccone F, Pekkarinen P.T, Friberg H, Cronberg T, Jakobsen J.C, Skrifvars M.B

**Affiliations:** ^1^ Emergency Medicine and Services, Helsinki University Hospital and University of Helsinki Helsinki Finland; ^2^ Department of Anaesthesia and Intensive Care University of Helsinki and Helsinki University Hospital Helsinki Finland; ^3^ Intensive Care Unit, Wellington Hospital Wellington New Zealand; ^4^ Medical Research Institute of New Zealand Wellington New Zealand; ^5^ Australian and New Zealand Intensive Care Research Centre, Monash University Melbourne Victoria Australia; ^6^ Department of Critical Care University of Melbourne Melbourne Victoria Australia; ^7^ Department of Clinical Sciences Lund Anesthesia and Intensive Care, Lund University Lund Sweden; ^8^ Department of Anesthesia and Intensive Care Helsingborg Hospital Helsingborg Sweden; ^9^ Adult Critical Care, University Hospital of Wales Cardiff UK; ^10^ Institute of Clinical Medicine University of Eastern Finland Kuopio Finland; ^11^ Department of Anesthesiology and Intensive Care Kuopio University Hospital Kuopio Finland; ^12^ Critical Care Program, the George Institute for Global Health, UNSW Sydney Australia; ^13^ Malcolm Fisher Department of Intensive Care Royal North Shore Hospital St Leonards Australia; ^14^ Neurology, Department of Clinical Sciences Lund Lund University Lund Sweden; ^15^ Department of Neurology Skåne University Hospital Lund Sweden; ^16^ Department of Clinical Sciences Lund Section of Cardiology, Skåne University Hospital Lund Sweden; ^17^ Tampere University Hospital, Wellbeing Services County of Pirkanmaa and Tampere University, Faculty of Medicine and Health Technology Tampere Finland; ^18^ Department of Clinical Sciences Lund Lund University Lund Sweden; ^19^ Department of Research, Development, Education and Innovation Skåne University Hospital Lund Sweden; ^20^ Department of Neurology and Rehabilitation Skåne University Hospital Lund Sweden; ^21^ Critical Care Division and Department of Intensive Care Medicine The George Institute for Global Health Sydney Australia; ^22^ St George Hospital Clinical School University of New South Wales Sydney Australia; ^23^ Department of Neurology Helsinki University Hospital and University of Helsinki Helsinki Finland; ^24^ Anesthesia & Intensive Care, Department of Clinical Sciences Lund University, Skane University Hospital Malmö Sweden; ^25^ Copenhagen Trial Unit, Centre for Clinical Intervention Research, Copenhagen University Hospital – Rigshospitalet Copenhagen Denmark; ^26^ Department of Regional Health Research Faculty of Health Sciences, University of Southern Denmark Copenhagen Denmark; ^27^ Intensive and Perioperative Care, Skåne University Hospital Malmö Sweden; ^28^ Intensive Care Unit Tampere University Hospital Tampere Finland; ^29^ Research Unit of Translational Medicine, Medical Research Center Oulu, Oulu University Hospital and University of Oulu Oulu Finland; ^30^ Critical Care Center and Research Group of Intensive Care Medicine, Oulu University Hospital, MRC Oulu and University of Oulu Oulu Finland; ^31^ Department of Clinical Sciences, Anesthesia and Intensive Care Lund University, Skåne University Hospital Malmö Sweden; ^32^ Department of Clinical Sciences Helsingborg Anesthesia and Intensive Care, Lund University Lund Sweden; ^33^ Department of Intensive and Perioperative Care Skåne University Hospital, Lund University Lund Sweden; ^34^ Department of Operation and Intensive Care Hallands Hospital Halmstad Halmstad Sweden; ^35^ Department of Anaesthesiology and Intensive Care Medicine Institute of Clinical Sciences, Sahlgrenska Academy, University of Gothenburg Gothenburg Sweden; ^36^ Perioperative Medicine and Intensive Care (PMI), Karolinska University Hospital Stockholm Sweden; ^37^ Department of Physiology and Pharmacology Karolinska Institutet Stockholm Sweden; ^38^ Department of Clinical Science, Intervention and Technology Karolinska Institute Stockholm Sweden; ^39^ North Estonia Medical Centre Tallinn Estonia; ^40^ Department of Anesthesia and Intensive Care Medicine, Division of Emergencies and Critical Care Oslo University Hospital Oslo Norway; ^41^ Lovisenberg Diaconal University College Oslo Norway; ^42^ Department of Intensive Care Medicine Stavanger University Hospital Stavanger Norway; ^43^ Department of Anaesthesia and Intensive Care Medicine Centre Hospitalier de Luxembourg Luxembourg Luxembourg; ^44^ Department of Life Sciences and Medicine Faculty of Science, Technology and Medicine, University of Luxembourg Esch‐sur‐Alzette Luxembourg; ^45^ University Heart Center Lübeck, University Hospital Schleswig‐Holstein Lübeck Germany; ^46^ German Center for Cardiovascular Research (DZHK), partner Site Hamburg/Lübeck/Kiel Lübeck Germany; ^47^ Department of Neurology and Stroke University Hospital Tuebingen Tuebingen Germany; ^48^ Hertie Institute of Clinical Brain Research Tuebingen Germany; ^49^ Department of Neurology Freie Universität and Humboldt‐Universität Zu Berlin, Charité—Universitätsmedizin Berlin Berlin Germany; ^50^ Department of Nephrology and Medical Intensive Care Charité ‐ Universitaetsmedizin Berlin Berlin Germany; ^51^ Department of Intensive Care Medicine Ghent University Hospital Ghent Belgium; ^52^ Department of Cardiology Ziekenhuis Oost‐Limburg Genk Belgium; ^53^ Wellcome‐Wolfson Institute for Experimental Medicine Queen's University Belfast Belfast UK; ^54^ Regional Intensive Care Unit Royal Victoria Hospital Belfast UK; ^55^ CEDAR (Centre for Healthcare Evaluation, Device Assessment and Research) Cardiff and Vale University Health Board Cardiff Cardiff UK; ^56^ Bristol Royal Infirmary, University Hospitals Bristol and Weston Bristol UK; ^57^ Guy's & St Thomas' Hospital London UK; ^58^ Intensive Care Medicine Centre for Human & Applied Physiological Sciences, School of Basic & Medical Biosciences Faculty of Life Sciences and Medicine King's College London UK; ^59^ Intensive Care Medicine, King's Critical Care, King's College Hospital, NHS Foundation Trust London UK; ^60^ Department of Perioperative Medicine Barts Heart Centre, St Bartholomew's Hospital London UK; ^61^ St George's University Hospital NHS Foundation Trust London UK; ^62^ Department of Critical Care Portsmouth University Hospitals Trust Cosham Portsmouth UK; ^63^ Leeds Teaching Hospitals NHS Trust Leeds UK; ^64^ University College Dublin Clinical Research Centre at St Vincent's University Hospital, University College Dublin Dublin Ireland; ^65^ The Alfred Hospital Melbourne Australia; ^66^ Institute of Intensive Care Medicine University Hospital Zurich Zurich Switzerland; ^67^ Medical Faculty, University of Zurich Zurich Switzerland; ^68^ Department of Intensive Care Medicine Inselspital University Hospital Bern Bern Switzerland; ^69^ Klinik für Intensivmedizin, Kantonsspital St. Gallen St. Gallen Switzerland; ^70^ Istituto Cardiocentro Ticino Lugano Switzerland; ^71^ Division of Intensive Care and Emergency Medicine, Department of Internal Medicine Medical University Innsbruck Innsbruck Austria; ^72^ AOM, IRCCS Policlinico San Martino Genoa Italy; ^73^ Dipartimento di Scienze Chirurgiche Diagnostiche Integrate University of Genova Genoa Italy; ^74^ 2nd Dept. of Internal Medicine, Cardiovascular Medicine General University Hospital and 1st Faculty of Medicine, Charles University in Prague Prague Czech Republic; ^75^ Institute for Heart Diseases, Wroclaw Medical University Wrocław Poland; ^76^ 2nd Department of Medicine, Department of Cardiovascular Medicine First Faculty of Medicine, Charles University in Prague and General University Hospital in Prague Prague Czech Republic; ^77^ King Saud Bin Abdulaziz University for Health Sciences and King Abdullah International Medical Research Center Riyadh Saudi Arabia; ^78^ Department of Anesthesia Critical Care Medicine, and Pain Medicine, Al‐Amiri Hospital, Ministry of Health Kuwait City Kuwait; ^79^ Kuwait Extracorporeal Life Support Program, Al‐Amiri Center for Respiratory and Cardiac Failure, Ministry of Health Kuwait City Kuwait; ^80^ Department of Anesthesia, Critical Care and Pain Medicine Jaber Alahmad Alsabah Hospital Kuwait City Kuwait; ^81^ Tan Tock Seng Hospital Singapore Singapore; ^82^ Yong Loo Lin School of Medicine, National University of Singapore Singapore Singapore; ^83^ Lee Kong Chian School of Medicine, Nanyang Technological University Singapore Singapore; ^84^ Intensive Care Unit, Liverpool Hospital, South Western Sydney Local Health District Sydney New South Wales Australia; ^85^ South Western Clinical School, University of New South Wales Sydney New South Wales Australia; ^86^ The Ingham Institute for Applied Medical Research Sydney New South Wales Australia; ^87^ Liverpool Hospital Sydney New South Wales Australia; ^88^ Department of Cardiology Royal Prince Alfred Hospital Sydney Australia; ^89^ Caboolture and Royal Brisbane and Women's Hospitals, Metro North Hospital and Health Service Brisbane Queensland Australia; ^90^ School of Clinical Medicine, Queensland University of Technology Brisbane Queensland Australia; ^91^ Critical Care Division, the George Institute for Global Health, University of New South Wales Sydney New South Wales Australia; ^92^ School of Medicine and Public Health, University of Newcastle Newcastle New South Wales Australia; ^93^ Intensive Care Unit, John Hunter Hospital Newcastle New South Wales Australia; ^94^ Northern Clinical School, Sydney Medical School, University of Sydney Sydney New South Wales Australia; ^95^ Department of Intensive Care Hospital Universitaire de Bruxelles (HUB), Universite Libre de Bruxelles (ULB) Brussels Belgium; ^96^ Anesthesia and Intensive Care, Department of Clinical Sciences Lund Lund University Lund Sweden

## Abstract

**Background:**

Acute kidney injury (AKI) is a common and serious complication after cardiac arrest, affecting more than 30% of initially successfully resuscitated patients, with around 10% receiving Kidney Replacement Therapy (KRT). Currently, treatment options to prevent renal complications are limited. In this predefined sub‐study of the Sedation, TEmperature and Pressure after Cardiac Arrest and REsuscitation (STEPCARE) trial, we evaluate the effects of sedation depth, fever management strategies, and mean arterial pressure (MAP) targets on the risk of major adverse kidney events (MAKE) in post‐cardiac arrest patients.

**Methods:**

STEPCARE‐MAKE is a predefined prospective sub‐study of the international, randomized clinical STEPCARE trial. The main trial will enroll 3500 patients and employs a 2 × 2 × 2 factorial design, randomizing participants across three interventions: (1) deep sedation for 36 h or minimal sedation, (2) fever management with or without a feedback‐controlled device, and (3) MAP target ≥ 65 mmHg or ≥ 85 mmHg. The primary outcome is the composite outcome MAKE, which includes death from any cause by day 30, initiation of KRT, or persistent renal dysfunction, defined as the final creatinine value ≥ 200% of the baseline at the time of discharge from the primary hospital or at day 30, whichever occurs first. Differences between baseline and the highest in‐hospital creatinine, baseline and the last measured in‐hospital creatinine, baseline and 72‐h creatinine, and baseline and the highest creatinine within 72 h will be reported as secondary outcomes.

**Conclusion:**

The findings of this sub‐study will provide new evidence on the renal effects of the STEPCARE trial interventions and may inform the future of individualized kidney‐protective treatment approaches.

## 1 Background

Among patients admitted to the intensive care unit (ICU) after initially successful resuscitation from cardiac arrest, a significant proportion develop a post–cardiac arrest syndrome leading to multiorgan dysfunction [1, 2]. Acute kidney injury (AKI) is a common and serious complication, affecting over 30% of patients after cardiac arrest, with kidney replacement therapy (KRT) initiated in 10%–13% of patients [3, 4, 5, 6, 7]. In patients resuscitated from cardiac arrest, both the occurrence and severity of AKI independently predict the development of chronic kidney disease and are associated with increased short‐ and long‐term mortality, particularly when renal recovery is incomplete [8, 9]. Even transient severe AKI has significant economic implications, and it adversely affects the quality of life of survivors [10, 11, 12, 13]. If chronic kidney disease develops, long‐term consequences become even more significant [14, 15, 16].

STEPCARE‐MAKE, a sub‐study of the Sedation, TEmperature and Pressure after Cardiac Arrest and REsuscitation (STEPCARE) trial, was designed to provide information on different strategies of sedation, fever control, and mean arterial pressure (MAP) targets with respect to major adverse kidney events (MAKE) in post‐cardiac arrest patients [17, 18, 19]. It will be the largest prospective trial evaluating relevant renal endpoints in relation to these interventions in critically ill patients resuscitated from out‐of‐hospital cardiac arrest (OHCA).

## 2 Methods

### 2.1 Trial Design

STEPCARE‐MAKE is a predefined sub‐study of the multicenter, international, academic, investigator‐initiated, parallel‐group, randomized superiority trial STEPCARE. This sub‐study aims to evaluate the incidence of MAKE in patients resuscitated from OHCA. The main trial employs a 2 × 2 × 2 factorial design in which every participant will be randomized to three comparisons: providing either continuous sedation for 36 h or minimal sedation, managing fever with or without a feedback‐controlled device, and targeting MAP ≥ 85 mmHg or ≥ 65 mmHg [17, 18, 19].

Each intervention will be evaluated independently with respect to the outcomes of this sub‐study, and the results will be reported in accordance with CONsolidated Standards Of Reporting Trials (CONSORT) guidelines [20, 21]. The full protocol of the STEPCARE trial is available at https://stepcare.org/protocol.

### 2.2 Inclusion and Exclusion Criteria

Data from all patients enrolled in the main STEPCARE trial will be used in the STEPCARE‐MAKE sub‐study, meaning that identical inclusion and exclusion criteria will be applied [17, 18, 19].

Eligible patients are adults (≥ 18 years old) with OHCA of non‐traumatic origin and sustained return of spontaneous circulation (ROSC). To be eligible, patients must be either unconscious or intubated and sedated due to agitation.

Patients with OHCA of traumatic or hemorrhagic origin, those with confirmed or suspected intracranial hemorrhage, patients receiving extracorporeal membrane oxygenation (ECMO) prior to randomization and pregnant patients will be excluded. Each patient may only be randomized once.

Detailed baseline and participant characteristics are provided in Tables S1, S5, and S10.

### 2.3 Screening and Randomization

Patient screening will be performed as part of the main STEPCARE trial and is described in detail elsewhere [17, 18, 19]. All patients initially successfully resuscitated from OHCA and admitted to ICU will be screened and randomized using permuted blocks of varying sizes.

### 2.4 Interventions, General Intensive Care, Prognostication and Withdrawal of Life‐Supporting Therapies (WLST)

The duration of the intervention is 36 h from randomization for sedation, and 72 h or until extubation, whichever occurs first, for interventions concerning fever management and MAP targets. The allocated fever management strategies and MAP targets will be reinstated in the event of reintubation during that period. After the intervention period, treatment will follow usual care at the discretion of the treating clinician.

For participants randomized to receive continuous sedation, a Richmond Agitation‐Sedation scale (RASS) score −4 to −5 will be targeted [22]. For those assigned to receive minimal sedation, weaning from all sedatives will be pursued as soon as possible, and thereafter sedatives will be administered only as needed for clinical care targeting a RASS score of 0 to −2. In both groups, short‐acting sedative agents should be preferred.

Device‐based fever management will be conducted using either an endovascular or surface cooling device with a feedback‐controlled system if a single core body temperature measurement reaches 37.8°C, with the target temperature set to 37.5°C. Fever management of participants assigned to receive temperature control without a device will follow standard ICU practice. Conservative measures to limit temperature rise will be allowed in both groups.

The methods (fluid therapy, vasopressors, and inotropes) used to achieve the assigned MAP targets of ≥ 65 mmHg or ≥ 85 mmHg will be at the treating clinician's discretion, in accordance with local practices.

Deviation from assigned targets will be documented, including the underlying reason. Acceptable reasons for target deviations are presented in Tables S2, S6, and S11, and cumulative medication doses in Tables S3, S8, and S12. The utilization and type of a cooling device (invasive or non‐invasive) is provided in Table S7. Mean MAP and bladder temperatures achieved are illustrated in Figures S1 and S3, and the proportion of participants in deep sedation (RASS −4 or −5) in Figure S2.

General care, including decisions to initiate KRT and cardiac interventions, will be managed consistently across all allocation groups, in accordance with local protocols and standardized care plans.

A conservative and standardized protocol for neurological prognostication, based on the recommendations of the European Resuscitation Council and European Society of Intensive Care Medicine, will be employed [23].

Detailed descriptions of the interventions, neurological prognostication, and withdrawal of life‐sustaining therapies applied are provided in the STEPCARE trial protocols [17, 18, 19]. https://stepcare.org/protocol.

### 2.5 Blinding

Due to the nature of the interventions, blinding of personnel directly involved in patient care, including those making decisions regarding KRT, is not feasible. However, measures will be taken to prevent the dissemination of allocation information outside this group.

### 2.6 Follow‐Up

After the intervention period, the highest and final creatinine value measured at the primary hospital will be recorded. According to the STEPCARE protocol, formal blinded follow‐ups are scheduled for 30 days and 6 months after cardiac arrest [17, 18, 19]. In STEPCARE‐MAKE, the information on vital status from the 30‐day follow‐up will be used.

### 2.7 Outcome Measures

The primary outcome of this sub‐study is a composite outcome of MAKE, defined as death from any cause by day 30, initiation of KRT during the stay in the primary hospital, or persistent renal dysfunction, defined as a final creatinine value ≥ 200% of the baseline at the time of discharge from the primary hospital or at day 30, whichever occurs first. Baseline creatinine is defined as the highest outpatient creatinine in the previous 6 months, or if unavailable, the creatinine on admission. Patients meeting any of these criteria will be considered to have reached the composite outcome.

The proportion of patients who received KRT and are still alive at day 30, and the proportion of patients with persistent renal dysfunction who are alive at day 30 and did not receive KRT during the ICU stay, will be reported separately as will the proportion of survivors with persistent renal dysfunction.

Differences between baseline and the highest in‐hospital creatinine, baseline and the last measured in‐hospital creatinine, baseline and 72‐h creatinine, and baseline and the highest creatinine within 72 h will be reported as secondary outcomes.

Detailed outcome measures for the different interventions are presented in Tables S4, S9, and S13.

### 2.8 Rationale for Chosen Outcomes

The composite outcome MAKE was first introduced by Shaw et al. in 2011 and has since been widely used in numerous randomized trials [24]. It is also recommended by the National Institute of Diabetes and Digestive and Kidney Disease (NIDDK) as a primary endpoint in phase three trials assessing kidney outcomes [25, 26].

Despite its limitations—including inequality between different components and heterogeneity in definition between previous trials—MAKE is a comprehensive and clinically meaningful primary endpoint [27]. All its components (death, new need for KRT, and persistent renal dysfunction) are highly relevant from both a patient‐centered perspective and in terms of healthcare resource use.

### 2.9 Data Collection and Management

STEPCARE‐MAKE uses data collected and managed as part of the main STEPCARE trial [17, 18, 19]. Information will be obtained from hospital records, relatives, and ambulance services. Detailed data on baseline characteristics, relevant medications, clinical status, and laboratory markers will be collected at enrollment, during ICU stay, at discharge, and at the 30 days follow‐up. All data is entered into a central electronic Case Report Form. Detailed descriptions of the collected data are provided in the Supporting Informations.

### 2.10 Sample Size and Power Estimation

This sub‐study is not independently powered; its sample size is determined by the main STEPCARE trial, with planned enrollment of 3500 patients. The main trial sample‐size calculation, based on data from the previous cardiac arrest trials TTM and TTM2, and the International Cardiac Arrest Registry (INTCAR), assuming an overall mortality of 60% in the control arm, was powered to detect a 5.6% absolute risk reduction in this primary endpoint [7, 28].

Based on the trials: Blood pressure and Oxygenation targets after out‐of‐hospital cardiac arrest (BOX), Therapeutic Hypothermia after Cardiac Arrest in Non‐shockable Rhythm (HYPERION) and Target Temperature Management After Cardiac Arrest (TTM), the anticipated incidence of AKI requiring KRT is approximately 10%–12% [5, 7, 29]. In the BOX‐trial, the survival rate among patients who received KRT during ICU stay was 43% [5]. Extrapolating from that data, if 12% of enrolled patients received KRT during their ICU stay and had a 43% survival, then 5% of all enrolled patients would be discharged alive after receiving KRT. In addition, we expect 1%–2% of patients who did not receive KRT during their ICU stay to have persistent renal dysfunction at discharge from the primary hospital. Based on these assumptions, we estimate that around 67% (60% + 5% + 2%) of patients would meet the composite primary endpoint during this trial.

Assuming a 67% incidence of the primary composite outcome in the control group, a significance level of 0.05 and 90% power, the available sample size allows detection of an absolute risk reduction in MAKE of 5.3% (from 67.0% to 61.7%) by any of these interventions in STEPCARE, corresponding to a relative risk reduction of 7.9%.

For initiation of KRT using the same significance level and statistical power, and assuming the incidence of 12%, an absolute risk reduction of 3.4% (28.3% relative risk reduction) could be detected.

### 2.11 Statistical Analysis

The primary outcome of the STEPCARE‐MAKE will be analyzed in the intention‐to‐treat population as a binary variable (event met or not) at 30 days. Analyses will be conducted using mixed‐effects generalized linear model. The analysis will be adjusted for site as a random effect and for the allocation of the other two STEPCARE interventions as fixed effects. Possible interactions between interventions will be assessed. Outcomes will be presented as the proportion of patients who reach the endpoint; risk ratios and absolute differences with 95% confidence intervals (CIs) will be calculated for all interventions separately. If there are statistically significant differences in the use of KRT between geographical areas (Europe/Oceania/Other) we will consider adjusting for these with an exploratory model with geographical area as a fixed effect.

An exploratory win ratio analysis will be conducted to compare outcomes between intervention and control groups. Each patient in the intervention group will be paired with each patient in the control group, and pairs will be evaluated according to a prespecified hierarchy based on the MAKE components [30]. Each patient in the intervention group will be paired with each patient in the control group, and pairs will be evaluated according to a prespecified hierarchy: (1) death within 30 days, (2) initiation of KRT, (3) persistent renal dysfunction, and (4) difference between baseline and the last measured creatinine before death or discharge from the primary hospital.

For each pair, comparison will begin with death. If one patient dies and the other survives, that survivor is considered the winner for the pair. If both patients survive, the next endpoint in the hierarchy will be evaluated. If one patient has a more favorable outcome, that patient is considered the winner; in case of a tie, the comparison goes on to the next endpoint. Pairs with no differences across all components will be classified as ties.

The win ratio will be calculated as the total number of wins divided by the total number of losses for the intervention group, with ties excluded. A 95% confidence interval for the win ratio will be estimated using established methods. As this analysis is exploratory, results will be interpreted descriptively.

Secondary outcomes—differences between baseline and the highest in‐hospital creatinine, baseline and the last in‐hospital creatinine, baseline and 72‐h creatinine as well as baseline and the highest creatinine within 72 h—will be analyzed as continuous variables using mixed‐effects linear regression. The analysis will be adjusted for site as a random effect and for the allocation of the other two STEPCARE interventions as fixed effects. Results will be presented as median values with interquartile ranges (IQR). The absolute and relative differences will be calculated, and 95% CI will be reported.

Missing data will be handled according to recommendations published by Jakobsen et al. [31] Statistical analysis of the main trial will be used in this sub‐study [32].

### 2.12 Subgroup Analysis

Analysis of the following subgroups will be performed:

– Age (≥/< 65 years).
– Sex at birth (male/female).
– Circulatory shock on admission (yes/no).
– History of hypertension with pharmacological treatment (yes/no).
– History of diabetes mellitus (yes/no).
– History of known severe chronic kidney disease (CKD4, eGFR<30) (yes/no).
– Baseline risk of poor functional outcome (Miracle2‐score: low risk [0–2], medium risk [3, 4, 5], and high risk [6, 7, 8, 9, 10]) [33].
– Any previous cardiac comorbidity (history of previous coronary artery bypass grafting [CABG], percutaneous coronary intervention [PCI], or heart failure with pharmacological treatment) (yes/no).
– Cardiac cause of arrest (yes/no).
– Emergency coronary angiography performed on admission (yes/no).

Pre‐specified subgroups analyzed are listed in Table S14, and the corresponding results are illustrated in Figure S4.

### 2.13 Ethics and Informed Consent

No separate ethical approval was required for this sub‐study, as it is covered by the approved main STEPCARE protocol [17, 18, 19]. Ethical approval and a delayed consent procedure were granted for the main trial.

### 2.14 Trial Status and Timeline

Participant enrollment began in August 2023 and is expected to be completed in 2026. Data will be analyzed, and the results of the main trial will be published after completion of the 6‐month follow‐up period of the last enrolled participant. The results of this sub‐study are planned to be published late 2027.

## 3 Discussion

This sub‐study of the STEPCARE trial will investigate the effects of different sedation and blood pressure targets, as well as fever treatment strategies, on MAKE in post‐cardiac arrest patients, with potential relevance to other critically ill populations.

After the introduction of targeted hypothermia for patients resuscitated from cardiac arrest, deep sedation has been administered to facilitate the required care. In the general ICU population, trials have shown benefits of lighter sedation in mechanically ventilated patients, including shorter duration of mechanical ventilation, shorter ICU and hospital lengths of stay, and, in one study, decreased mortality [34, 35, 36]. However, complete avoidance of sedation does not seem to add any benefit compared to light sedation [37]. Two of those trials, one comparing light sedation to no sedation and one comparing light sedation to standard sedation, reported the incidence of AKI but found no between‐group differences [34, 37]. A post hoc analysis of a small pilot trial demonstrated significantly lower urine output in the light sedation group compared with no sedation, but no statistically significant differences in the incidence of AKI (based on creatinine values) or in the need for KRT [38]. The effects of intentional deep sedation have not previously been compared to the lightest possible in terms of MAKE.

Previous trials evaluating temperature control in patients resuscitated from cardiac arrest have mainly focused on comparing normothermia to targeted hypothermia and the duration of that treatment. Some of these trials have evaluated renal outcomes as secondary endpoints or as adverse events but have not identified differences in the need for KRT or in the incidence of milder stages of AKI [7, 29, 39, 40, 41]. Whether active fever prevention has beneficial effects on kidney function remains largely unknown. Pharmacological fever control in the general ICU population has been evaluated in randomized trials. Both paracetamol and ibuprofen appear to provide only a modest decrease in body temperature and have been neutral with respect to renal adverse events and mortality [42, 43, 44]. Active fever control with external cooling devices in non‐neurological patients has been assessed only in small studies with heterogeneous results, and only a portion of these studies have reported renal outcomes. A significant reduction in the need for KRT in the device‐based cooling group was found in the French SEPSISCOOL‐I pilot‐study of 200 patients comparing the treatment of fever in patients with sepsis with or without a cooling device [45]. The REACTOR pilot‐study of 184 febrile unselected mechanically ventilated patients in the ICU also showed a nonsignificant trend towards reduction in the need for KRT in the group receiving active temperature management [46]. No significant difference was instead found in creatinine values in a small Chinese study [47]. A larger prospective SEPSISCOOL‐II trial aiming to recruit 820 participants evaluating device‐based fever prevention in septic and febrile ICU patients is yet to be published [48]. Given the large sample size of the current STEPCARE‐MAKE study, the findings will have ramifications on AKI prevention strategies in critical illness.

The optimal MAP target remains unclear despite several studies across different populations, target levels, and scenarios, and so far, no clear benefit of any strategy has been demonstrated. Four large, randomized trials have evaluated the effects of different MAP targets on renal outcomes, three of those in septic patients, and one in post‐cardiac arrest patients with conflicting results. The SEPSISPAM trial of 776 septic patients found no difference in kidney function between the two groups in general with MAP targets of 65–70 mmHg or 80–85 mmHg, but in the subgroup of previously hypertensive patients, a significantly lower proportion of patients received KRT or experienced doubling of plasma creatinine in the higher MAP group [49]. A recent Japanese OPTPRESS trial of 518 patients 65 years or older with septic shock with the same blood pressure targets was terminated early on the basis of an interim analysis suggesting higher mortality and less KRT‐free days at day 28 in the higher‐target group [50]. The 65 trial of 2600 older patients (65 years or older) with vasodilatory shock found no difference in either the need for KRT or mortality when comparing MAP targets 60–65 mmHg to over 65 mmHg, but the difference in MAP between the groups was minimal and no subgroup analysis comparing renal outcomes was done [51]. The largest blood pressure study in patients resuscitated from OHCA, the BOX trial, reported no difference in the need for KRT between MAP targets of 63 mmHg and 77 mmHg in 789 enrolled patients [52].

A small trial in septic cirrhotic patients and a post hoc analysis of trials on patients resuscitated from OHCA (COMACARE and NEUROPROTECT) demonstrated no difference in relevant kidney outcomes between different MAP targets [53, 54]. Three recent meta‐analyses with heterogeneous critically ill patient populations, published before the OPTPRESS trial, demonstrated no difference in the need for KRT or in milder degrees of AKI between higher and lower MAP targets, but they did not analyze specific subgroups [55, 56, 57].

### 3.1 Strengths and Limitations

Key strengths of the STEPCARE‐MAKE trial include its large, international, multicenter design with few exclusion criteria, allowing robust statistical precision and generalizability across the population resuscitated from OHCA. The prospective nature and predefined and clinically meaningful outcomes further strengthen the study.

The study has several limitations. The sample size, although large, was determined by the power calculations of the main STEPCARE trial, and no additional adjustments could be made specifically for this sub‐study. In addition, the components of the primary composite endpoint, while all clinically relevant, differ in clinical importance; given the high predicted overall mortality in this population—largely related to WLST mostly due to neurological injury—even small differences in mortality may dominate the composite outcome and outweigh less frequent renal events.

Further considerations relate to measurement of renal function. Baseline creatinine values may not always be available in outpatient records, and admission values may already be influenced by pre‐arrest pathophysiology, potentially leading to an underestimation of the true baseline renal function. Furthermore, discharge or interhospital transfer policies may vary geographically, affecting the interval between admission and the last in‐hospital creatinine measurement.

Indications for initiating KRT are determined by local practice rather than standardized criteria and may therefore vary between clinicians, centers, and countries. The treating clinicians making the decisions to initiate KRT are unblinded to the allocation groups, and the specific reasons for initiating KRT will not be collected. In addition, urine output data are not collected as part of this trial, making classification of AKI according to the Kidney Disease: Improving Global Outcome (KDIGO) criteria impossible [58].

Finally, some patients may have been receiving chronic renal replacement therapy prior to enrollment, although the proportion has been low in previous trial, only 0.5% [52]. Because this information is not collected, it is possible that such patients could have a minor influence on the results, as their distribution across allocation groups remains unknown.

The factorial design of the main trial also poses the challenge of not being able to completely rule out interactions between interventions on the measured outcomes.

## 4 Conclusion

This prospective sub‐study of a randomized clinical trial may provide evidence on the effects of the three critical care interventions—depth of sedation, fever management, and blood pressure targets—on MAKE after out‐of‐hospital cardiac arrest.

## Author Contributions

Keski‐Keturi M. drafted the original manuscript. Other authors contributed to the study design, critically reviewed the manuscript, and approved the final version.

## Funding

The STEPCARE trial, including this sub‐study, is funded by multiple research councils and foundations from several countries: the Swedish Research Council, ALF‐project funding within Swedish Health Care, Grants from the South Swedish Health Region, The Academy of Finland, Finska Läkaresällskapet, Sigrid Juselius Stiftelse, Medicinska Understödsföreningen Liv och Hälsa, Svenska Kulturfonden, Stiftelsen Dorothea Olivia, Karl Walter och Jarl Walter Perkléns minne, Medical Research Future Fund (Australia), Health Research Council of New Zealand, the Clinical Research Programme, Directorate of Health, Ministry of Health and Social Security, Luxembourg, and the Fondation Coeur—Daniel Wagner, Luxembourg.

## Conflicts of Interest

Leithner C. received research support from Laerdal Foundation. Nee J. received honorarium and travel costs for presentations from BD BARD and Xenios AG. Skrifvars M. B. is a member of the editorial board of Acta Anaesthesiologica Scandinavica. Hästbacka J. has received a consultation fee from Paion (2022) and she is a member of the editorial board of Acta Anaesthesiologica Scandinavica.

## Supporting information


**Data S1:** Supporting Information.
**Table S1:** Characteristics of participants in sedation intervention*.
**Table S2:** Reasons for deviation from the allocated sedation target.
**Table S3:** Cumulative doses of medications between groups.
**Table S4:** Outcomes of sedation intervention.
**Table S5:** Characteristics of participants in temperature intervention*.
**Table S6:** Reasons for deviation from the allocated temperature targets.
**Table S7:** Use of a cooling device.
**Table S8:** Cumulative doses medications between groups.
**Table S9:** Outcomes of temperature intervention.
**Table S10:** Characteristics of participants in MAP intervention*.
**Table S11:** Reason for deviation from allocated MAP‐target.
**Table S12:** Cumulative doses of medications between groups.
**Table S13:** Outcomes of MAP intervention.
**Table S14:** Pre‐specified subgroups of all intervention.
**Figure S1:** MAP of the higher and lower MAP target groups.
**Figure S2:** Proportion of RASS −4 or −5 by hour and in deep and no‐sedation group.
**Figure S3:** Mean temperatures of the groups with and without a device‐based fever management.
**Figure S4:** Pre‐specified subgroups.

## Data Availability

Data sharing not applicable to this article as no datasets were generated or analysed during the current study.
