# Supplementary material for: Effects of Sedation, TEmperature and Pressure After Cardiac Arrest and REsuscitation on Major Adverse Kidney Events (STEPCARE‐MAKE): A Protocol for a Pre‐Planned Sub‐Study of a Randomized Clinical Trial
Source: Acta Anaesthesiol Scand. 2026 Jun 15;70(6):e70276. doi: 10.1111/aas.70276 (PMC13269662; doi:10.1111/aas.70276)

Effects of Sedation, TEmperature and Pressure after Cardiac Arrest and REsuscitation on Acute Kidney Injury (STEPCARE-AKI): a protocol for a pre-planned substudy of a randomized clinical trial

Supplementary information

Table of contents

[Data Collection 2](#_Toc216458398)

[Baseline data 2](#_Toc216458399)

[Prehospital data 3](#_Toc216458400)

[Background data 3](#_Toc216458401)

[Data on hospital admission 4](#_Toc216458402)

[Data in the intensive care unit 5](#_Toc216458403)

[At ICU discharge 6](#_Toc216458404)

[At hospital discharge 7](#_Toc216458405)

[30 days after randomization 7](#_Toc216458406)

[Table 1. Characteristics of participants in sedation intervention * 7](#_Toc216458407)

[Table 2. Reasons for deviation from the allocated sedation target 9](#_Toc216458408)

[Table 3. Cumulative doses of medications between groups 9](#_Toc216458409)

[Table 4. Outcomes of sedation intervention 10](#_Toc216458410)

[Table 5. Characteristics of participants in temperature intervention* 11](#_Toc216458411)

[Table 6. Reasons for deviation from the allocated temperature targets 12](#_Toc216458412)

[Table 7. Use of a cooling device 12](#_Toc216458413)

[Table 8. Cumulative doses medications between groups 13](#_Toc216458414)

[Table 9. Outcomes of temperature intervention 13](#_Toc216458415)

[Table 10. Characteristics of participants in MAP intervention* 14](#_Toc216458416)

[Table 11. Reason for deviation from allocated MAP-target 16](#_Toc216458417)

[Table 12. Cumulative doses of medications between groups 16](#_Toc216458418)

[Table 13. Outcomes of MAP intervention 17](#_Toc216458419)

[Table 14. Pre-specified subgroups of all intervention 18](#_Toc216458420)

[Mock-figure 1. MAP of the higher and lower MAP target groups 19](#_Toc216458421)

[Mock-figure 2. Proportion of RASS -4 or -5 by hour and in deep and no-sedation group 20](#_Toc216458422)

[Mock-Figure 3. Mean temperatures of the groups with and without a device-based fever management 21](#_Toc216458423)

[Forest plot figure 1. Pre-specified subgroups 22](#_Toc216458424)

## Data Collection

### Baseline data

- Pre-randomization characteristics:
  - Inclusion and exclusion criteria
  - Age
  - Sex at birth
  - Time and date of Cardiac arrest
  - Time and date of return of spontaneous circulation (ROSC)
- MIRACLE2 -score and its components:
  - Witnessed / Unwitnessed arrest
  - Shockable initial rhythm (yes/no)
  - Reactive pupils at ROSC (yes/no)
  - Age (under 60/60-80/over 80 years)
  - Changing rhythm (any two of the following ventricular fibrillation (VF), pulseless electrical activity (PEA), or asystole) (yes/no)
  - pH below 7.2 (yes/no)
  - Epinephrine given (yes/no)
- Presumed cause of arrest:
  - Cardiac – ST-elevation myocardial infarction (STEMI) / acute coronary occlusion
  - Cardiac – non-ST-elevation myocardial infarction (NSTEMI)
  - Cardiac – Arrhythmia nor related to acute myocardial ischemia
  - Cardiac – Heart failure
  - Cardiac – Other cardiac cause
  - Pulmonary embolism
  - Hypoxia
  - Other medical cause (electrolyte disorder, sepsis etc.)
  - Asphyxia (strangulation, foreign body etc.)
  - Drowning
  - Drug overdose
  - Trauma/Bleeding
  - Intracranial bleeding
- Perceived prognosis by randomizing physician (good or poor neurological function)

### Prehospital data

- Scene of cardiac arrest (home, work, public, nursing facility, ambulance, other)
- Witnessed arrest (yes/no)
- Bystander cardiopulmonary resuscitation (CPR) (yes/no)
- First monitored rhythm at arrival of emergency medical services (asystole / PEA / VF / non-perfusing ventricular tachycardia (VT) / ROSC after bystander defibrillation / unknown)
- Time of emergency call
- Minutes until CRP started (no-flow time)

### Background data

- Height
- Weight
- Pre-arrest:
  - Functional status (independent / non-independent in daily life)
  - Frailty using the Clinical Frailty Score (1-9)
- Previous history of:
  - Percutaneous coronary intervention (PCI) (yes/no)
  - Coronary artery bypass grafting (CABG) (yes/no)
  - Heart failure with pharmacological treatment (yes/no)
  - Implantable cardioverter defibrillator (ICD) (yes/no)
  - Hypertension with pharmacological treatment (yes/no)
  - Diabetes mellitus (type 1 or type 2) (DM) (yes/no)
  - Stroke or transitory ischemic attack (yes/no)
  - Chronic obstructive pulmonary disease (COPD) (yes/no)
  - Severe chronic kidney disease (CKD4, eGFR <30) (yes/no)

### Data on hospital admission

- Time of ICU admission (day, hour)
- First recorded tympanic temperature (bilateral, highest value)
- Full Outline of UnResponsiveness (FOUR) motor score
- Preserved pupillary reflexes (yes/no)
- Preserved corneal reflexes (yes/no)
- First lactate (arterial or venous)
- First creatinine
- Highest outpatient (not in-hospital) creatinine measured during the pervious 6 months prior to this cardiac arrest
- First troponin
- STEMI – New ST-segment elevation ≥1mm in ≥2 contiguous ECG leads or posterior STEMI
- ECG rhythm (sinus, atrial fibrillation or flutter, other)
- Shock on admission (systolic blood pressure < 90mmHg for at least 30 minutes or the need for supportive measures to maintain a systolic blood pressure ≥90mmHg and end-organ hypoperfusion (cool extremities, or urine output of less than 30ml/h, and a HR heart rate over 60 beats per minute)
- Severity of shock (SCAI class) (Beginning, Classic, Deteriorating, Extremis)
- Echocardiography performed during first 24h (yes/no)
- Left ventricular ejection fraction (LVEF) (normal > 55%, mildly reduced 40-54%, moderately reduced 30-39%, severely reduced < 30%)
- Depressed right ventricular function (yes/no)
- Other pathology (severe aortic stenosis, severe mitral regurgitation, severe tricuspid regurgitation, regional wall motion abnormality, none)

### Data in the intensive care unit

- At 0, 2, 4, 6, 8, 10, 12, 14, 16, 18, 20, 22, 24, 28, 32, 36, 40, 48, 56, 72, 96, and 120 hours:
  - Core temperature (bladder, rectal, or esophageal)
  - Systolic, diastolic, and mean arterial pressures
  - Heart rate
- At 0, 4, 8, 12, 16, 20, 24, 28, 32, 36, 40, 48, 72, 96, and 120 hours:
  - Richmond Agitation-Sedation Scale (RASS) score
  - Propofol dose (mg/kg/h)
  - Dexmedetomidine dose (mcg/kg/h)
  - Noradrenaline dose (mcg/kg/min)
  - Midazolam infusion (yes/no)
  - Dobutamine infusion (yes/no)
  - Adrenaline infusion (yes/no)
  - Responds commands (yes/no)
- At 0, 12, 24, 36, 48, 72, 96, and 120 hours:
  - Mechanically ventilated (yes/no)
  - Respiratory rate (/min)
  - Oxygen saturation (arterial, measured by blood gas)
  - Fraction of inspired oxygen
  - Arterial partial pressure of oxygen
  - Arterial partial pressure of carbon dioxide
  - pH
  - Peak end-expiratory pressure
  - Lactate (arterial)
  - Tidal volume
  - Ventilator mode:
    - Pressure control
    - Volume control
    - Pressure support
    - Not done / extubated
  - FOUR motor score
  - Corneal reflexes present (yes/no)
  - Pupillary reflexes present (yes/no)
  - Highest BedSide Shivering Assessment Scale score
  - Any tonic clonic seizure activity since the last time point (yes/no)
  - Any status myoclonus since last time point (yes/no)
  - Highest ICU mobility score during the last 24 hours (0-10)
  - Delirium present (assessed by Confusion Assessment Method for the ICU (CAM-ICU) or Intensive Care Delirium Screening Checklist (ICDSC)) during the last 24 hours (yes/no)
- At 0, 24, 48, 72, 96, 120
- At 72 hours:
  - Cumulative doses of:
    - Noradrenaline
    - Propofol
    - Midazolam
    - Dexmedetomidine
    - Remifentanil
    - Fentanyl
    - Sufentanil
    - Oxycodone
    - Morphine
    - Paracetamol/acetaminophen

### At ICU discharge

- Time of ICU discharge
- Last LVEF measured (normal, or mildly, moderately, or severely reduced)
- Date, time and value of the highest measured cardiac troponin
- Readmission to ICU (yes/no)
- Time and results of coronary angiography if performed (1-vessel, 2-vessle, 3-vessel disease)
  - Culprit lesion found on coronary angiography (yes/no)
    - Acute thrombotic culprit occlusion (yes/no)
    - Location of culprit lesion (LM, LAD, Cx, RCA or graft)
  - PCI performed (yes/no)
- CABG performed (yes/no)
- ICD implanted before leaving the hospital (yes/no)
- Use of mechanical cardiac support and the time of initiation
  - Impella (or another percutaneous ventricular assist device)
  - ECMO
  - Intra-aortic balloon pump (IABP)
- Use of haloperidol, olanzapine, or quetiapine (yes/no)
- Use of antiseizure medication (yes/no)
- Discharge facility (Coronary care unit / general ward / other ICU / dead)
- Use and type of temperature control device (yeas/no/type)
- Continuous sedation for the entire 36 hours post-randomization (yes/no)
- Time of final extubation (or is still intubated)
- Time of awakening
- Highest creatinine during ICU-stay
- Renal replacement therapy (RRT) (yes/no)
- Safety events
- Neurological prognosis performed (yes/no)
  - Date and time
  - Confounding factors ruled out (yes/no)
  - Hours from the last dose of sedative agents at the beginning of prognostication
  - FOUR motor score at the timepoint of prognostication
  - Corneal or pupillary reflexes bilaterally absent at the timepoint of prognostication (yes/no)
  - Myoclonic status < 72 hours post arrest
  - Reason for not performing the prognostication at 27 hours
- Withdrawal of life-sustaining therapies (yes/no)

### At hospital discharge

- Date and time of hospital discharge as obtained from hospital notes or registries
- Discharged to: nursing home / rehabilitation unit / other hospital / home / dead)
- Death (yes/no)
  - Date and time
  - Presumed cause (cerebral, cardiac, multi-organ failure, brain death, other)
- Probable cause of cardiac arrest
- Last in-hospital creatinine
- Patient still on dialysis when discharged from primary hospital (yes/no)
- Additional data on observational parameters (including, but not limited to data on circulatory indices, EEG, imaging, pupillometry etc.)

### 30 days after randomization

- Vital status (dead / alive)

| Table 1. Characteristics of participants in sedation intervention * | **Continuous sedation**  **(N=x)** | **Minimal sedation**  **(N=x)** |
| --- | --- | --- |
| Demographic characteristics |  |  |
| Age (years) |  |  |
| Male sex at birth, no (%) |  |  |
| Medical history, no. (%) |  |  |
| Estimated pre-arrest functional status |  |  |
| Independent in basic activities of life |  |  |
| Dependent in basic activities of life |  |  |
| Percutaneous coronary intervention |  |  |
| Coronary artery bypass grafting |  |  |
| Heart failure with pharmacologic treatment |  |  |
| Implantable cardioverter defibrillator  (ICD) |  |  |
| Hypertension with pharmacologic treatment |  |  |
| Stroke or transitory ischemic attack |  |  |
| Chronic obstructive pulmonary disease (COPD) |  |  |
| Diabetes mellitus |  |  |
| Severe chronic kidney disease† |  |  |
| Baseline out-patient creatinine (mmol/l) £ |  |  |
| Baseline creatinine on admission (mmol/l) |  |  |
| Characteristics of cardiac arrest, no. (%) |  |  |
| Scene of cardiac arrest |  |  |
| Home |  |  |
| Work |  |  |
| Public Place |  |  |
| Nursing facility |  |  |
| Ambulance |  |  |
| Other |  |  |
| Bystander witnessed cardiac arrest |  |  |
| Bystander performed cardiopulmonary resuscitation |  |  |
| Changing rhythms (any 2 of VF/PEA/asystole) |  |  |
| Adrenaline administered‡ |  |  |
| Pupillary reflexes present bilaterally prior randomization, no. (%) |  |  |
| First monitored rhythm, no. (%) |  |  |
| Ventricular fibrillation |  |  |
| Ventricular tachycardia |  |  |
| ROSC after bystander-initiated defibrillation |  |  |
| Unknown shockable rhythm |  |  |
| Pulseless electrical activity |  |  |
| Asystole |  |  |
| Unknown non-shockable rhythm |  |  |
| Median time from cardiac arrest to initiation of advanced life support, min (IQR) |  |  |
| Median time from cardiac arrest to ROSC, min (IQR) |  |  |
| Median time from ROSC to randomization, min (IQR) |  |  |
| Clinical characteristics at hospital admission: |  |  |
| First recorded temperature (◦C) |  |  |
| First pH |  |  |
| Clinical characteristics at ICU admission |  |  |
| FOUR motor score § |  |  |
| Corneal reflexes present bilaterally, no. (%) |  |  |
| Pupillary reflexes present bilaterally, no. (%) |  |  |
| Circulatory shock, no. (%) ¶ |  |  |
| ST-segment elevation myocardial infarction ─ no. (%) |  |  |
| *VF*, ventricular fibrillation, *PEA*, pulseless electrical activity, *IQR* interquartile range, and *ROSC* return of spontaneous circulation.  *Some of these data may be presented in tables in the supplement  †Estimated glomerular filtration rate <30 ml/min/1.73 m^2^  £Defined as the highest outpatient creatinine six months prior to admission  ‡Prior ROSC and hospital arrival by emergency medical service  §Full Outline of Unresponsiveness (FOUR) motor scores range from 0 to 4, with higher scores indicating better motor function  ¶Shock on admission is defined as a systolic blood pressure of less than 90 mmHg for at least 30 min or the need for supportive measure to maintain a systolic blood pressure ≥ 90 and end organ hypoperfusion (cool extremities, or urine output of less than 30 ml/hr and heart rate >60/min | | |

| Table 2. Reasons for deviation from the allocated sedation target | |
| --- | --- |
| **Direction of deviation** | **Reason for deviation** |
| Lighter sedation | To allow for assessment of brain death |
| Lighter sedation | Other reasons |
| Deeper sedation | To facilitate intensive care unit care |
| Deeper sedation | Shivering |
| Deeper sedation | Seizures/myoclonus |
| Deeper sedation | Delirium/agitation |
| Deeper sedation | Other reasons |

| Table 3. Cumulative doses of medications between groups | | | | |
| --- | --- | --- | --- | --- |
| **Cumulative doses up to 72 hours** | **Continuous sedation N=X** | **Minimal sedation N=X** | **Relative risk (95% CI)** | **p-value** |
| Propofol (mg) |  |  |  |  |
| Dexmedetomidine (mg) |  |  |  |  |
| Midazolam (mg) |  |  |  |  |
| Oxycodone (mg) |  |  |  |  |
| Morphine (mg) |  |  |  |  |
| Fentanyl |  |  |  |  |
| Remifentanil (mg) |  |  |  |  |
| Noradrenalin (mg) |  |  |  |  |

| Table 4. Outcomes of sedation intervention | | | | |
| --- | --- | --- | --- | --- |
| **Outcome** | **Continuous sedation**  **N =X** | **Minimal sedation**  **N=X** | **Relative risk (95% CI)** | **Absolute risk difference (95% CI)** |
| **Primary outcome** |  |  |  |  |
| MAKE, no. (%) *¥ |  |  |  |  |
| **Components of primary outcome** |  |  |  |  |
| Death from any cause at day 30, no. (%) |  |  |  |  |
| Renal replacement therapy, no. (%) |  |  |  |  |
| Among survivors no./total no. (%) |  |  |  |  |
| Persistent renal dysfunction defined as final creatinine value ≥200% of the baseline value, no. (%) ¥ |  |  |  |  |
| Among survivors, no./total no. (%) |  |  |  |  |
| Among survivors without renal-replacement therapy, no./total no. (%) |  |  |  |  |
| **Secondary outcomes** |  |  |  |  |
| Difference between baseline and highest in-hospital creatinine, median (IQR) (µmol/l) ¥ |  |  |  |  |
| Difference between baseline and the last measured in-hospital creatinine, median (IQR) (µmol/l) ¥ |  |  |  |  |
| Difference between baseline and 72-hour creatinine, median (IQR) (µmol/l) ¥ |  |  |  |  |
| Difference between baseline and the highest creatinine within 72 hours, median (IQR) (µmol/l) ¥ |  |  |  |  |
| *Composite endpoint of death from any cause, renal replacement therapy, or persistent renal dysfunction (defined as final creatinine value ≥ 200% of the baseline value) at day 30 or hospital discharge, whichever came first  ¥Baseline creatinine is defined as the highest outpatient creatinine in the previous six months, or if unavailable, the creatinine on admission | | | | |

| Table 5. Characteristics of participants in temperature intervention* | **Fever management without a device**  **(N=x)** | **Fever management with a device**  **(N=x)** |
| --- | --- | --- |
| Demographic characteristics |  |  |
| Age (years) |  |  |
| Male sex, no (%) |  |  |
| Medical history, no. (%) |  |  |
| Estimated pre-arrest functional status |  |  |
| Independent in basic activities of life |  |  |
| Dependent in basic activities of life |  |  |
| Percutaneous coronary intervention |  |  |
| Coronary artery bypass grafting |  |  |
| Heart failure with pharmacologic treatment |  |  |
| Implantable cardioverter defibrillator |  |  |
| Hypertension with pharmacologic treatment |  |  |
| Stroke or transitory ischemic attack |  |  |
| Chronic obstructive pulmonary disease (COPD) |  |  |
| Diabetes mellitus |  |  |
| Severe chronic kidney disease† |  |  |
| Baseline out-patient creatinine (mmol/l) £ |  |  |
| Baseline creatinine on admission (mmol/l) |  |  |
| Characteristics of cardiac arrest, no. (%) |  |  |
| Scene of cardiac arrest |  |  |
| Home |  |  |
| Work |  |  |
| Public Place |  |  |
| Nursing facility |  |  |
| Ambulance |  |  |
| Other |  |  |
| Bystander witnessed cardiac arrest |  |  |
| Bystander performed cardiopulmonary resuscitation |  |  |
| Changing rhythms (any 2 of VF/PEA/asystole) |  |  |
| Adrenaline administered‡ |  |  |
| Pupillary reflexes present bilaterally prior randomization, no. (%) |  |  |
| First monitored rhythm, no. (%) |  |  |
| Ventricular fibrillation |  |  |
| Ventricular tachycardia |  |  |
| ROSC after bystander-initiated defibrillation |  |  |
| Unknown shockable rhythm |  |  |
| Pulseless electrical activity |  |  |
| Asystole |  |  |
| Unknown non-shockable rhythm |  |  |
| Median time from cardiac arrest to initiation of advanced life support, min (IQR) |  |  |
| Median time from cardiac arrest to ROSC, min (IQR) |  |  |
| Median time from ROSC to randomization, min (IQR) |  |  |
| Clinical characteristics at hospital admission: |  |  |
| First recorded temperature (◦C) |  |  |
| First pH |  |  |
| Clinical characteristics at ICU admission |  |  |
| FOUR motor score § |  |  |
| Corneal reflexes present bilaterally, no. (%) |  |  |
| Pupillary reflexes present bilaterally, no. (%) |  |  |
| Circulatory shock, no. (%) ¶ |  |  |
| ST-segment elevation myocardial infarction ─ no. (%) |  |  |
| *VF*, ventricular fibrillation, *PEA*, pulseless electrical activity, *IQR* interquartile range, and *ROSC* return of spontaneous circulation  *Some of these data may be presented in tables in the supplement  †Estimated glomerular filtration rate <30 ml/min/1.73 m^2^ calculated from the creatinine value  £Defined as the highest outpatient creatinine during six months prior to admission or if unavailable, cretinine on admission  ‡Prior ROSC and hospital arrival by emergency medical service  §Full Outline of Unresponsiveness (FOUR) motor scores range from 0 to 4, with higher scores indicating better motor function  ¶Shock on admission is defined as a systolic blood pressure of less than 90 mmHg for at least 30 min or the need for supportive measure to maintain a systolic blood pressure ≥ 90 and end organ hypoperfusion (cool extremities, or urine output of less than 30 ml/h and heart rate >60/min | | |

| Table 6. Reasons for deviation from the allocated temperature targets | |
| --- | --- |
| **Direction of deviation** | **Reason for deviation** |
| Using device | Severe reaction to pharmacological agents |
| Using device | Unsuitable for NSAID or acetaminophen |
| Using device | Other reasons |
| Avoiding device | Skin complication due to the device (surface device) |
| Avoiding device | Infection due to the device (intravascular) |
| Avoiding device | Bleeding or thrombosis due to the device (intravascular) |
| Avoiding device | Other reasons |

| Table 7. Use of a cooling device | **Fever management with a device**  **N=X** | **Fever management without a device**  **N=X** |
| --- | --- | --- |
| Type of device |  |  |
| Invasive device, no. (%) |  |  |
| Non-invasive device, no (%) |  |  |
| Total, no (%) |  |  |

| Table 8. Cumulative doses of medications between groups | | | | |
| --- | --- | --- | --- | --- |
| **Cumulative doses up to 72 hours** | **Fever management with a device N=X** | **Fever management without a device N=X** | **Relative risk (95% CI)** | **p-value** |
| Propofol (mg) |  |  |  |  |
| Dexmedetomidine (mg) |  |  |  |  |
| Midazolam (mg) |  |  |  |  |
| Oxycodone (mg) |  |  |  |  |
| Morphine (mg) |  |  |  |  |
| Fentanyl |  |  |  |  |
| Remifentanil (mg) |  |  |  |  |
| Noradrenaline (mg) |  |  |  |  |

| Table 9. Outcomes of temperature intervention | | | | |
| --- | --- | --- | --- | --- |
| **Outcome** | **Fever management with a device**  **N=X** | **Fever management without a device**  **N=X** | **Relative risk (95% CI)** | **Absolute risk difference (95% CI)** |
| **Primary outcome** |  |  |  |  |
| MAKE, no. (%) * |  |  |  |  |
| **Components of primary outcome** |  |  |  |  |
| Death from any cause at day 30, no. (%) |  |  |  |  |
| Renal replacement therapy, no. (%) |  |  |  |  |
| Among survivors no./total no. (%) |  |  |  |  |
| Persistent renal dysfunction defined as final creatinine value ≥200% of the baseline value, no. (%) ¥ |  |  |  |  |
| Among survivors, no./total no. (%) |  |  |  |  |
| Among survivors without renal-replacement therapy, no./total no. (%) |  |  |  |  |
| **Secondary outcomes** |  |  |  |  |
| Difference between baseline and highest in-hospital creatinine, median (IQR) (µmol/l) ¥ |  |  |  |  |
| Difference between baseline and the last measured in-hospital creatinine, median (IQR) (µmol/l) ¥ |  |  |  |  |
| Difference between baseline and 72-hour creatinine, median (IQR) (µmol/l) ¥ |  |  |  |  |
| Difference between baseline and the highest creatinine within 72 hours, median (IQR) (µmol/l) ¥ |  |  |  |  |
| * Composite endpoint of death from any cause, renal replacement therapy, or persistent renal dysfunction (defined as final creatinine value ≥ 200% of the baseline value) at day 30 or hospital discharge, whichever came first  ¥ Baseline creatinine is defined as the highest outpatient creatinine in the previous six months, or if unavailable, the creatinine on admission | | | | |

| Table 10. Characteristics of participants in MAP intervention* | **MAP >65mmHg**  **(N=x)** | **MAP >85mmHg**  **(N=x)** |
| --- | --- | --- |
| Demographic characteristics |  |  |
| Age (years) | x | x |
| Male sex, no (%) | x | x |
| Medical history, no. (%) |  |  |
| Estimated pre-arrest functional status |  |  |
| Independent in basic activities of life | x | x |
| Dependent in basic activities of life | x | x |
| Percutaneous coronary intervention | x | x |
| Coronary artery bypass grafting | x | x |
| Heart failure with pharmacologic treatment | x | x |
| Implantable cardioverter defibrillator | x | x |
| Hypertension with pharmacologic treatment | x | x |
| Stroke or transitory ischemic attack | x | x |
| Chronic obstructive pulmonary disease (COPD) | x | x |
| Diabetes mellitus | x | x |
| Severe chronic kidney disease† | x | x |
| Baseline out-patient creatinine (mmol/l) £ | x | x |
| Baseline creatinine on admission (mmol/l) | x | x |
| Characteristics of cardiac arrest, no. (%) |  |  |
| Scene of cardiac arrest |  |  |
| Home | x | x |
| Work | x | x |
| Public Place | x | x |
| Nursing facility | x | x |
| Ambulance | x | x |
| Other | x | x |
| Bystander witnessed cardiac arrest | x | x |
| Bystander performed cardiopulmonary resuscitation | x | x |
| Changing rhythms (any 2 of VF/PEA/asystole) | x | x |
| Adrenaline administered‡ | x | x |
| Pupillary reflexes present bilaterally prior randomization, no. (%) | x | x |
| First monitored rhythm, no. (%) |  |  |
| Ventricular fibrillation | x | x |
| Ventricular tachycardia | x | x |
| ROSC after bystander-initiated defibrillation | x | x |
| Unknown shockable rhythm | x | x |
| Pulseless electrical activity | x | x |
| Asystole | x | x |
| Unknown non-shockable rhythm | x | x |
| Median time from cardiac arrest to initiation of advanced life support, min (IQR) | x | x |
| Median time from cardiac arrest to ROSC, min (IQR) | x | x |
| Median time from ROSC to randomization, min (IQR) | x | x |
| Clinical characteristics at hospital admission: |  |  |
| First recorded temperature (◦C) | x | x |
| First pH | x | x |
| Clinical characteristics at ICU admission |  |  |
| FOUR motor score § | x | x |
| Corneal reflexes present bilaterally, no. (%) | x | x |
| Pupillary reflexes present bilaterally, no. (%) | x | x |
| Circulatory shock, no. (%) ¶ | x | x |
| ST-segment elevation myocardial infarction ─ no. (%) | x | x |
| *VF*, ventricular fibrillation, *PEA*, pulseless electrical activity, *IQR* interquartile range, and *ROSC* return of spontaneous circulation.  *Some of these data may be presented in a table/tables in the supplement  †Estimated glomerular filtration rate <30 ml/min/1.73 m^2^  £Defined as the highest outpatient creatinine six months prior to admission  ‡Prior ROSC and hospital arrival by emergency medical service  §Full Outline of Unresponsiveness (FOUR) motor scores range from 0 to 4, with higher scores indicating better motor function  ¶Shock on admission is defined as a systolic blood pressure of less than 90 mmHg for at least 30 min or the need for supportive measure to maintain a systolic blood pressure ≥ 90 and end organ hypoperfusion (cool extremities, or urine output of less than 30 ml/hr and heart rate >60/min | | |

| Table 11. Reason for deviation from allocated MAP-target | |
| --- | --- |
| **Direction of deviation** | **Reason of deviation** |
| Lower target | Not achieving the targeted MAP despite vasoactive escalation |
| Lower target | Cardiac reasons* |
| Lower target | Magor surgery |
| Lower target | Intracranial bleeding |
| Lower target | Extracranial bleeding |
| Lower target | Other reasons |
| Lower target | Clinical team human error |
| Higher target | Clinical team human error |
| Higher target | Suspected inadequate renal perfusion |
| Higher target | Ischemic stroke or critical carotid stenosis |
| Higher target | Other reasons |
| *Severe arrythmias, worsening pulmonary oedema, worsening cardiogenic shock, left ventricular outflow tract obstruction, aortic insufficiency, or other clinically | |

| Table 12. Cumulative doses of medications between groups | | | | |
| --- | --- | --- | --- | --- |
| **Cumulative doses up to 72 hours** | **MAP >65mmHg N=X** | **MAP >85mmHg N=X** | **Relative risk (95% CI)** | **p-value** |
| Propofol (mg) |  |  |  |  |
| Dexmedetomidine (mg) |  |  |  |  |
| Midazolam (mg) |  |  |  |  |
| Oxycodone (mg) |  |  |  |  |
| Morphine (mg) |  |  |  |  |
| Fentanyl |  |  |  |  |
| Remifentanil (mg) |  |  |  |  |
| Noradrenalin (mg) |  |  |  |  |

| Table 13. Outcomes of MAP intervention | | | | |
| --- | --- | --- | --- | --- |
| **Outcome** | **MAP > 65 mmHg**  **N=X** | **MAP > 85 mmHg**  **N=X** | **Relative risk (95% CI)** | **Absolute risk difference (95% CI)** |
| **Primary outcome** |  |  |  |  |
| MAKE, no. (%) *¥ |  |  |  |  |
| **Components of primary outcome** |  |  |  |  |
| Death from any cause at day 30, no. (%) |  |  |  |  |
| Renal replacement therapy, no. (%) |  |  |  |  |
| Among survivors no./total no. (%) |  |  |  |  |
| Persistent renal dysfunction defined as final creatinine value ≥200% of the baseline value, no. (%) ¥ |  |  |  |  |
| Among survivors, no./total no. (%) |  |  |  |  |
| Among survivors without renal-replacement therapy, no./total no. (%) |  |  |  |  |
| **Secondary outcomes** |  |  |  |  |
| Difference between baseline and highest in-hospital creatinine, median (IQR) (µmol/l) ¥ |  |  |  |  |
| Difference between baseline and the last measured in-hospital creatinine, median (IQR) (µmol/l) ¥ |  |  |  |  |
| Difference between baseline and 72-hour creatinine, median (IQR) (µmol/l) ¥ |  |  |  |  |
| Difference between baseline and the highest creatinine within 72 hours, median (IQR) (µmol/l) ¥ |  |  |  |  |
| * Composite endpoint of death from any cause, renal replacement therapy, or persistent renal dysfunction (defined as final creatinine value ≥ 200% of the baseline value) at day 30 or hospital discharge, whichever came first  ¥ Baseline creatinine is defined as the highest outpatient creatinine in the previous six months, or if unavailable, the creatinine on admission | | | | |

| Table 14. Pre-specified subgroups of all intervention |
| --- |
| Age (≥/< 65 years) |
| Sex at birth (male/female) |
| Circulatory shock on admission (yes/no) £ |
| History of hypertension with pharmacological treatment (yes/no) |
| History of diabetes mellitus (yes/no) |
| History of severe chronic kidney disease (CKD4, eGFR < 30 ml/min/1.73m2) (yes/no) |
| Baseline risk of poor functional outcome (Miracle2-score: low risk [0–2], medium risk [3-5], and high risk [6-10]) |
| Any previous cardiac comorbidity (history of previous coronary artery bypass grafting [CABG], percutaneous coronary intervention [PCI], or heart failure with pharmacological treatment) (yes / no) |
| Cardiac cause of arrest (yes/no) |
| Emergency coronary angiography performed on admission (yes/no) |
| *£ Systolic blood pressure <90mmHg for at least 30 minutes or vasopressor requirement to achieve a systolic blood pressure >90mmHg and signs of end organ hypoperfusion (Urine output less than 30 ml/h, cool extremities or heart rate >60/min)* |

##

## Mock-figure 1. MAP of the higher and lower MAP target groups


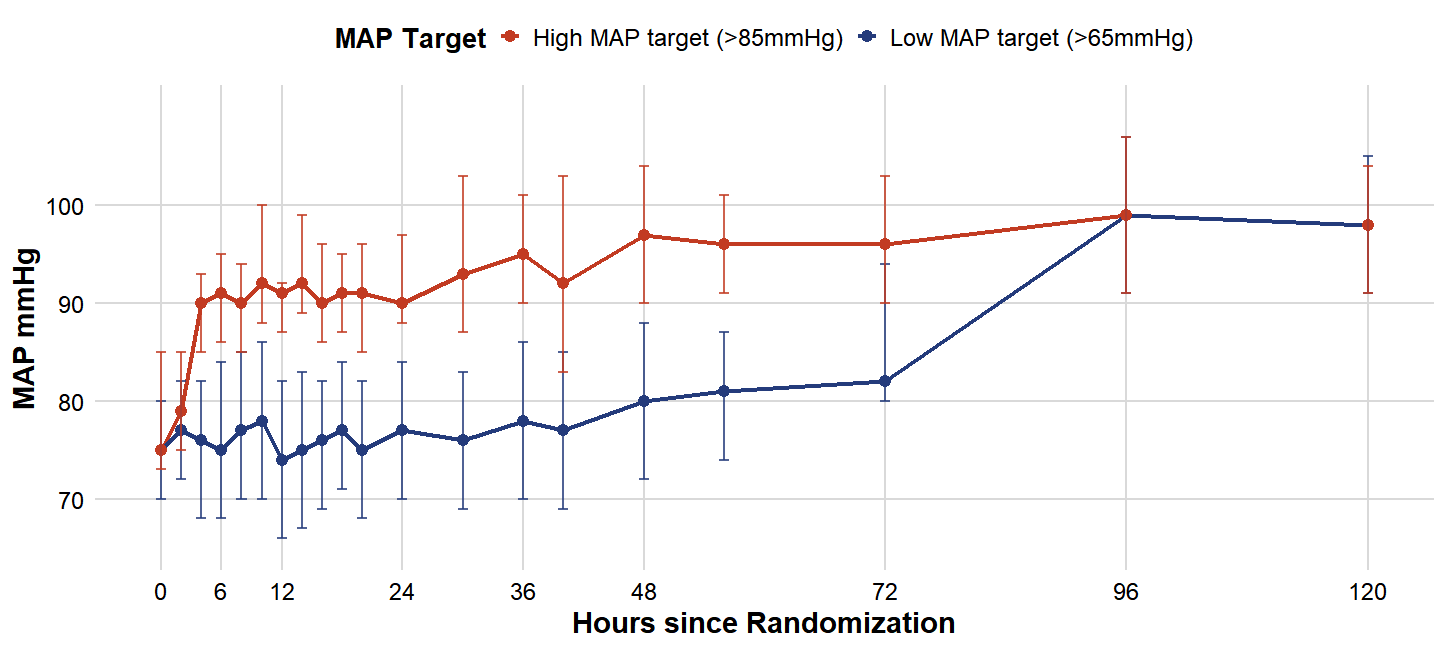


Mock-figure 1. MAP of the higher and lower MAP target groups, error bars indicate 2 standard deviations.

## Mock-figure 2. Proportion of RASS -4 or -5 by hour and in deep and no-sedation group


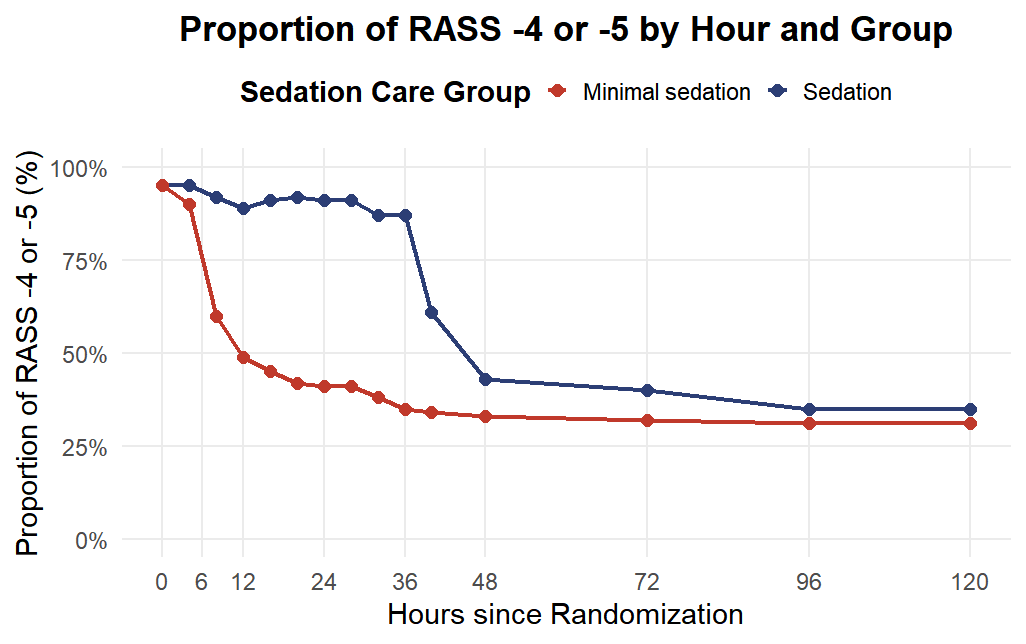


Mock-figure 2. Proportion of RASS -4 or -5 by hour and in deep and no-sedation group

## Mock-Figure 3. Mean temperatures of the groups with and without a device-based fever management
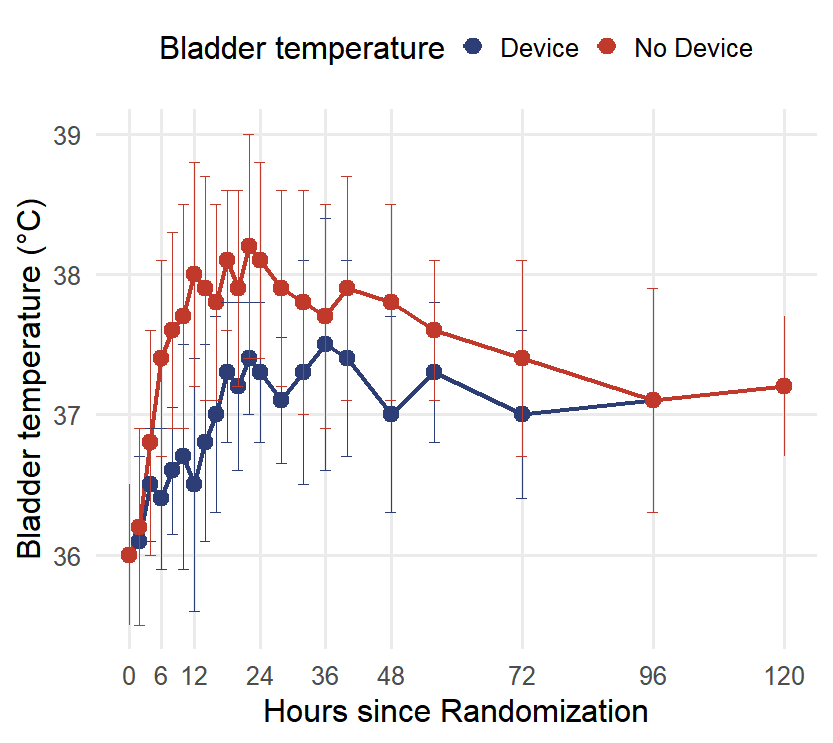


Mock-Figure 3. Mean temperatures of the groups with and without a device-based fever management, error bars indicate 2 standard deviations.

## Forest plot figure 1. Pre-specified subgroups


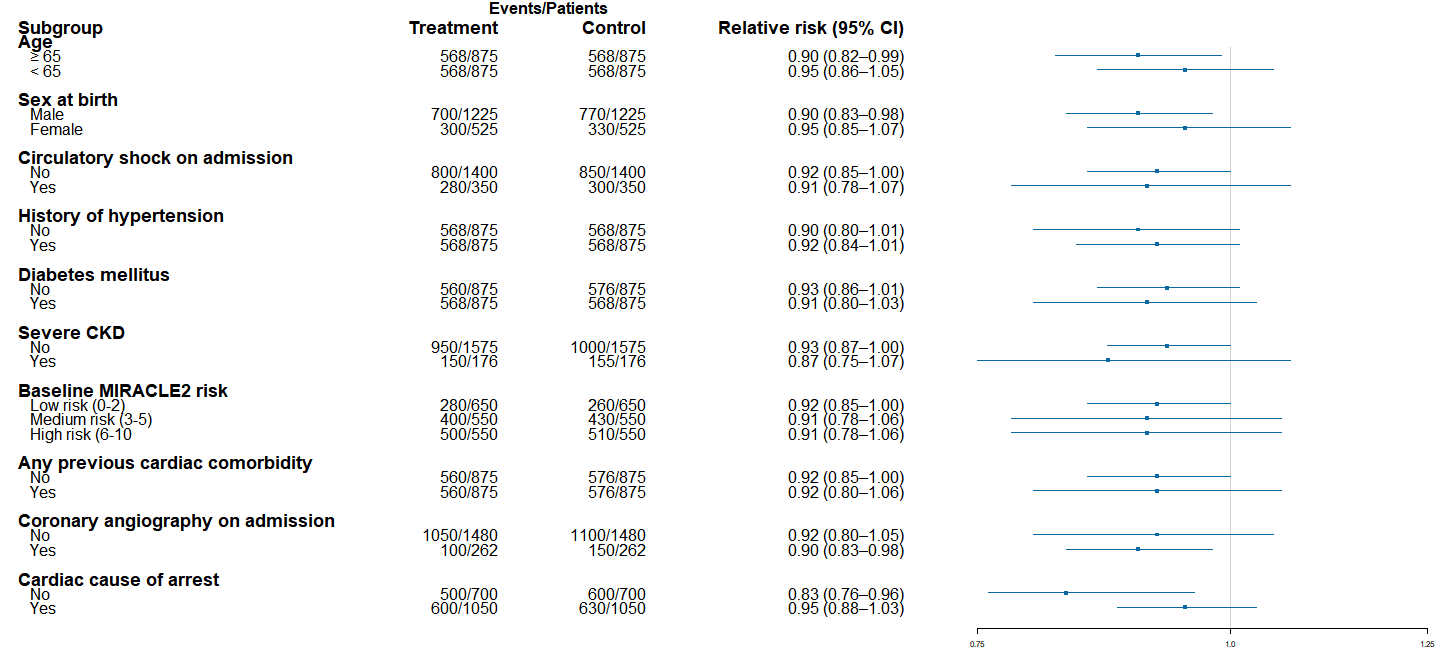

Supplement: Supplementary file 1 — Data S1: Supporting Information. Table S1: Characteristics of participants in sedation intervention*. Table S2: Reasons for deviation from the allocated sedation target. Table S3: Cumulative doses of medications between groups. Table S4: Outcomes of sedation intervention. Table S5: Characteristics of participants in temperature intervention*. Table S6: Reasons for deviation from the allocated temperature targets. Table S7: Use of a cooling device. Table S8: Cumulative doses medications between groups. Table S9: Outcomes of temperature intervention. Table S10: Characteristics of participants in MAP intervention*. Table S11: Reason for deviation from allocated MAP‐target. Table S12: Cumulative doses of medications between groups. Table S13: Outcomes of MAP intervention. Table S14: Pre‐specified subgroups of all intervention. Figure S1: MAP of the higher and lower MAP target groups. Figure S2: Proportion of RASS −4 or −5 by hour and in deep and no‐sedation group. Figure S3: Mean temperatures of the groups with and without a device‐based fever management. Figure S4: Pre‐specified subgroups. [file AAS-70-0-s001.docx]
